# Care Bundle for Acute Kidney Injury in Cardiac Patients: A Cluster-Randomized Trial

**DOI:** 10.3390/jcm12196391

**Published:** 2023-10-06

**Authors:** Ragna Iwers, Veronika Sliziuk, Michael Haase, Sophie Barabasch, Michael Zänker, Christian Butter, Anja Haase-Fielitz

**Affiliations:** 1 Department of Cardiology, Heart Center Brandenburg Bernau & Faculty of Health Sciences (FGW) Brandenburg, Brandenburg Medical School (MHB) Theodor Fontane, Ladeburger Str. 17, 16321 Bernau bei Berlin, Germany; ragna.iwers@gmail.com (R.I.); christian.butter@immanuelalbertinen.de (C.B.); 2 Institute of Social Medicine and Health System Research, Otto von Guericke University Magdeburg, 39120 Magdeburg, Germany; 3 Department of Cardiovascular Surgery, Heart Center Brandenburg Bernau & Faculty of Health Sciences (FGW) Brandenburg, Brandenburg Medical School (MHB) Theodor Fontane, 16321 Bernau bei Berlin, Germany; 4 Medical Faculty, Otto-von-Guericke University Magdeburg, 39106 Magdeburg, Germany; michael.haase@med.ovgu.de; 5 Diamedikum MVZ, 14473 Potsdam, Germany; 6 Department of Nephrology and Hypertension, Hannover Medical School, 30625 Hannover, Germany; 7 Department of Anesthesia and Intensive Care, Unfallkrankenhaus Berlin, 12683 Berlin, Germany; 8 Department of Gastroenterology & Internal Medicine, Heart Center Brandenburg Bernau & Faculty of Health Sciences (FGW) Brandenburg, Brandenburg Medical School (MHB) Theodor Fontane, 16321 Bernau bei Berlin, Germany

**Keywords:** acute kidney injury, AKI electronic alert system, care bundle, randomized, cardiac patients

## Abstract

Detection and timely intervention of acute kidney injury (AKI) is a major challenge worldwide. Electronic alerts for AKI may improve process- and patient-related endpoints. The present study evaluated the efficacy of an AKI electronic alert system and care bundle. This is a two-arm, prospective, cluster-randomized, controlled trial enrolling patients with AKI (KDIGO criteria) and cardiac diseases. Patients were randomly assigned to a routine care group or intervention group (DRKS-IDDRKS00017751). Two hundred patients (age 79 years, 46% female) were enrolled, with 100 patients in each group. The primary endpoint did not differ between patients in the routine care group 0.5 (−7.6–10.8) mL/min/1.73 m^2^ versus patients in the intervention group 1.0 (−13.5–15.1) mL/min/1.73 m^2^, *p* = 0.527. Proportions of patients in both study groups with hyperkalemia, pulmonary edema, and renal acidosis were comparable. The stop of antihypertensive medication during hypotensive periods was more frequent in patients in the intervention group compared to patients in the control group, *p* = 0.029. The AKI diagnosis and text module for AKI in the discharge letter were more frequently documented in patients in the intervention group (40%/48% vs. 25%/34%, *p* = 0.034; *p* = 0.044, respectively). Continued intake of RAAS inhibitors and the presence of a cardiac device were independently associated with a less pronounced decrease in eGFR from admission to the lowest value. In this RCT, electronic alerts for AKI and a care bundle improved process- but not patient-related endpoints.

## 1. Introduction

Acute kidney injury (AKI) is one of the most serious and common complications affecting inpatient admissions [1]. Early detection and appropriate management of AKI are vital to aid kidney recovery and to prevent related adverse outcomes [2]. A report by the National Confidence Enquiry into Patient Outcome and Death (NCEPOD) found that only 50% of patients with AKI received the appropriate standard of care [3]. In an observation study among survivors of AKI, about 80% of patients were not informed about AKI or nephrotoxic medications [4]. Electronic alerts in the hospitals were recommended to improve the recognition of AKI, and an AKI electronic alert system was mandated by NHS England in all Laboratory Information Management Systems across the NHS in 2015 [5]. Several trials focusing on patient safety, specialist referral, and clinical management showed positive effects of AKI electronic alert systems, including more frequent medication-related recommendations per patient, a reduced progression to higher AKI stage, emergency readmission to hospital, reduced length of stay in hospital and death during admission [6,7,8], see Appendix A, Table A1. A pragmatic stepped wedge cluster randomized trial showed improved AKI recognition, increased care, and shorter length of hospital stay in the intervention group but did not reduce 30-day AKI mortality [9]. However, differences in local context must be considered when study findings are interpreted. Specifically, in the UK, laboratory parameters from both the outpatient and inpatient settings are available to any treating physician at any time, enabling the detection of community-acquired and hospital-acquired AKI. Therefore, related study findings may not be transferable to regions in the world where laboratory parameters, such as serum creatinine, are not available cross-sectorally, and the patient is not being informed about their AKI. 

In a cluster-randomized, controlled study, we investigated if an AKI electronic alert system and care bundle would be able to improve process- and patient-related endpoints of patients with AKI. 

## 2. Materials and Methods 

### 2.1. Setting and Design

This is a two-arm prospective, cluster-randomized, controlled trial to evaluate the efficacy of an AKI electronic alert system and care bundle for hospitalized patients with AKI. The study was conducted at the Department of Cardiology, Heart Center Brandenburg Bernau, University Hospital of the Brandenburg Medical School (MHB), Germany, between January 2019 and March 2022, including a follow-up observation over 3 months regarding renal function and 12 months regarding survival status. 

Patients were assigned to the routine care or intervention group using cluster-randomized allocation, alternating at 4-week intervals. The sequence of allocation was specified prior to the start of the study using a random number generator.

This study is registered with the German Clinical Trial Register (DRKS-IDDRKS00017751). Ethical approval of the study protocol was obtained from the local ethics committee of the Brandenburg Medical School (E-01-20181101). 

### 2.2. Patients

To be included in the study, patients admitted to the Department of Cardiology had to have evidence of renal insufficiency according to KDIGO criteria [10], be over 18 years of age, and be able to sign a written informed consent form. KDIGO criteria were applied as creatinine increase (hospital-acquired AKI) or creatinine decrease (community-acquired AKI). Hospital-acquired AKI was defined as 0.3 mg/dL or 50% increase, respectively, in serum creatinine between hospital admission and highest serum creatinine within 48 h or 7 days, respectively. To be defined as community-acquired AKI, patients had to have, on admittance to the hospital or within 48 h of admission, a 33% decrease in serum creatinine from baseline within 7 days.

Patients who had already undergone kidney transplantation or who were receiving chronic or acute renal replacement therapy at the time of the study, pregnant patients, patients with HI/Hepatitis virus infections, or patients with a life expectancy below 3 months were excluded. 

### 2.3. Care Bundle (Intervention Group)

Prior to the study, an algorithm to detect relevant changes in serum creatinine was developed and embedded in the hospital information system. AKI was detected by an electronic alert system based on the Kidney Disease: Improving Global Outcomes (KDIGO) AKI criteria [10]. In addition, a notification system was added to the hospital’s SAP work system in the form of a lamp icon to indicate critical patient features and alert the ward physician to the problem of AKI. 

When a patient with AKI was identified, the study investigator received an internal email with information about the patient’s identity, department, creatinine increase, and the AKI stage, forwarding this information to the ward physician. In educational activities before study initiation, physicians were made aware of the AKI lamp icon, risk factors for AKI, and the KDIGO AKI care bundle to prevent further renal function deterioration and support renal recovery.

Patients in the intervention group were interviewed in detail by a medical colleague, their pre-existing conditions and risk factors were assessed, and their current medication regimen was checked. Measures of the care bundle [10] included the identification of the cause(s) of AKI in the clinical context, measures to achieve euvolemia (through fluid administration or negative balance), pharmacological intervention including discontinuation of nephrotoxic drugs or switch to less nephrotoxic drugs of the same substance class or monitoring of plasma levels, and the adjustment of drug doses to renal function. Also, optimizing hemodynamics, detection, and treatment of electrolyte and acid-base disorders, as well as monitoring of heart and kidney function during the index hospital stay (including blood pressure, heart rate, serum creatinine, diuresis, weight, and fluid balance) and nephrology consultation of patients with AKI stage 3 were part of the care bundle. Additionally, all patients received an information flyer and a kidney passport to record, together with the general practitioner, renal function values, enabling monitoring of these parameters. The passport could also be used to be presented in other medical institutions, such as pharmacies, to advise on alternatives to nephrotoxic medications. Finally, the discharge letter included information about participation in the study, AKI cause and severity, advice on subsequent kidney function tests in the outpatient setting three and twelve months after AKI, avoidance of NSAID, and sick day advice. 

### 2.4. Routine Care Group

In the study control period, patients were also contacted by the investigator—but later in the course of AKI—primarily to ask for permission to collect data on renal function within 3 months after AKI. In addition, in patients of the routine care group, a lamp icon in the hospital’s SAP work system (SAP Inc., Boston, MA, USA) indicated potential AKI. Patients in the routine care group received standard care for AKI, including the possibility of the ward physician consulting a nephrologist. 

### 2.5. Outcomes

The primary outcome was the loss of kidney filtration function from hospital admission to three months after AKI (change of eGFR). Secondary outcomes were the length of stay in the hospital, peak serum creatinine during an index hospital stay, AKI complications (hyperkalemia, kidney-related pulmonary edema, and renal acidosis), chronic renal replacement therapy, major adverse cardiac events (MACE) and rehospitalization within 90 days of the index admission as well as 3- and 12-month mortality.

### 2.6. Data Collection

Patient demographics and hospital data, comorbidities, medications, serum creatinine values during the index hospital stay, and other laboratory values were collected. 

The following variables were collected when available: admission serum creatinine (baseline), peak serum creatinine, and discharge serum creatinine, as well as serum creatinine 3 months after discharge. Discharge serum creatinine was defined as serum creatinine measured nearest to the date of hospital discharge. Urine output was not available. 

Also, the cause of AKI and AKI-related complications were documented. The surrogate marker of AKI management included information about AKI in the discharge letter (text module and AKI cause) and recommendations for outpatient follow-up. Three months after study enrolment, the primary care physician was contacted, and intercurrent renal function, need for chronic renal replacement therapy, rehospitalization within 90 days of the index admission, and possible cardiovascular events (MACE) were recorded. MACE was defined as acute myocardial infarction (AMI), stroke, or cardiovascular death.

### 2.7. Statistical Analysis 

Using data from a published randomized controlled trial [11], we estimated that 86 patients per group would be needed to have a 90% power to detect an absolute difference of 5 mL eGFR loss of patients in the intervention group between hospital admission and 3 months after discharge compared to the control group at a two-sided test with an alpha of 0.05 and a standard deviation of 10 mL eGFR. Assuming a 15% loss to follow-up in the primary endpoint, we aimed to enroll 100 patients per group. 

A Mann–Whitney U test was used for non-parametric two-group comparisons. The chi-squared test and Fisher’s exact test were used for dichotomous variables for two groups. Multivariable linear regression analysis for the decrease in GFR from admission to the lowest value included clinically relevant variables affecting eGFR. Patients with missing data (missing follow-up data or similar) were excluded from further examination. We report values as median with 25th to 75th percentiles or as a proportion of patients (%) as appropriate. A planned subgroup analysis for patients with community-acquired AKI was performed. Posthoc, additional subgroup analyses were performed for patients with diabetes, female gender, patients aged >70 years, patients with a cardiac device, or those using ACE inhibitors/AT-1 blockers.

A two-tailed *p*-value of <0.05 was defined as significant. Analysis was performed using SPSS version 27 (SPSS Inc., IBM, Chicago, IL, USA).

## 3. Results

### 3.1. Patient Characteristics

**Figure 1:**
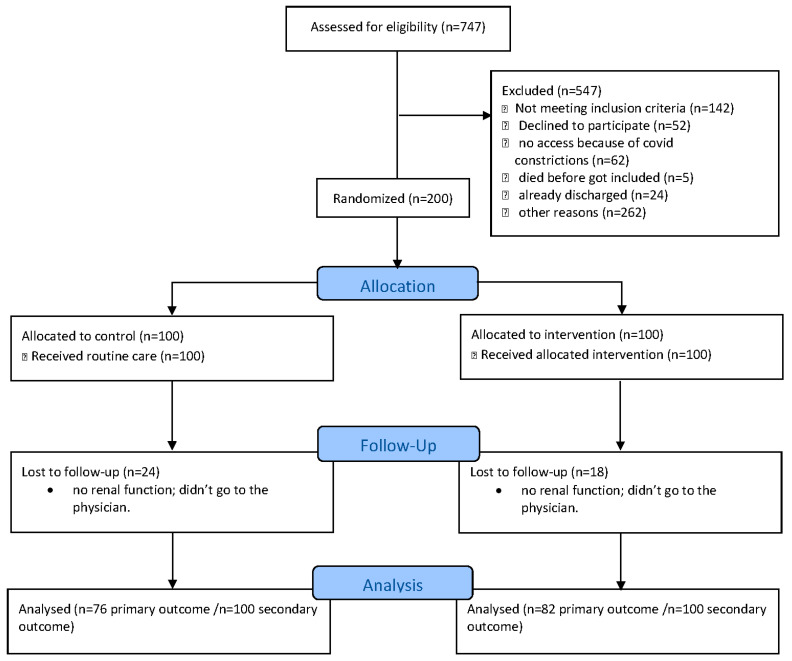
Flow chart.

Two hundred patients (aged 79 (68–84) years, 46% female) were prospectively randomized to a routine care group or intervention group. Patient flow is shown in Figure 1. The study groups were similar in terms of demographics, laboratory parameters, and most comorbidities (Table 1, Appendix A, Table A2). A higher proportion of patients in the routine care group presented with atrial fibrillation and were admitted electively compared to those in the intervention group. In the intervention group, more patients were admitted with acute coronary syndrome compared to patients in the routine care group (Table 1). Also, more patients in the routine care group received peri-interventional antibiotics (Table 2). 

### 3.2. Interventions

The proportions of patients receiving heart- and kidney-function-related interventions, including fluid administration, stopping potentially nephrotoxic medications, and nephrology consultation, were similar in both groups. However, stopping antihypertensive medication during hypotensive periods was more frequent in patients in the intervention group compared to patients in the control group (6% vs. 0%, *p* = 0.029); see Table 3.

### 3.3. Characteristics of AKI

**Figure 2:**
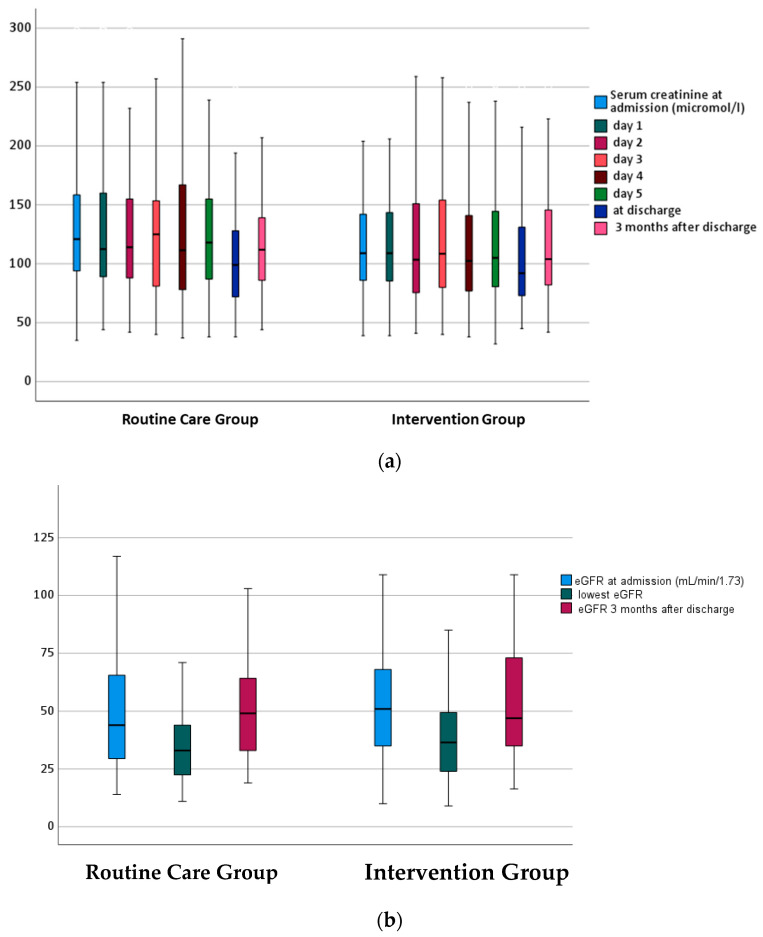
(**a**) Median serum creatinine concentration over time in both study groups. (**b**) Median eGFR concentration at admission, lowest during hospitalization, and 3 months after discharge in both study groups.

The course of serum creatinine and eGFR was similar in the study groups (Figure 2a,b). Loss of eGFR from admission to the lowest value was similar in both study groups (−8 vs. −9 mL/min/1.73 m^2^, *p* = 0.586, Table 4). In the routine care group, 59 patients had community-acquired AKI and 41 hospital-acquired AKI, and in the intervention group, 54 and 46 patients, respectively, *p* = 0.476. The most frequent cause of AKI was pre-renal, with 92% in the routine care group and 83% in the intervention group (Table 4). The severity of AKI was mostly stage 1, with no group differences (Table 1). In the routine care group, the AKI electronic alert was at 4 (2–8) days after hospital admission compared to 3 (2–8) days in the intervention group, *p* = 0.244. 

Also, the proportion of patients in both study groups with AKI-related complications, including hyperkalemia, pulmonary edema, and renal acidosis, were comparable (Table 5).

### 3.4. Primary Outcome

Change of eGFR from hospital admission to three months after AKI did not differ between patients in the routine care group 0.5 (−7.6–10.8) mL/min/1.73 m^2^ versus patients in the intervention group 1.0 (−13.5–15.1) mL/min/1.73 m^2^, *p* = 0.527 (Table 4). Also, three months after discharge, eGFR was similar in patients in the routine care group (49.1 mL/min/1.73 m^2^) compared to those in the intervention group (47.0 mL/min/1.73 m^2^), *p* = 0.770 (Figure 2b, Table 4). 

### 3.5. Process-Related Endpoints

**Figure 3:**
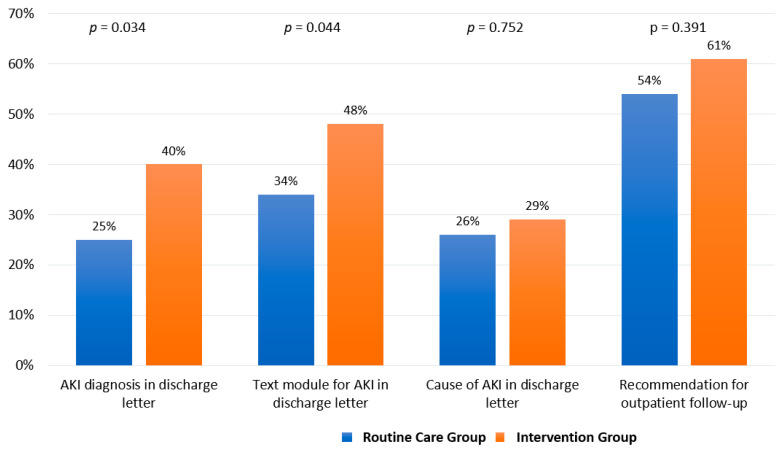
Process-related parameters.

In patients of the intervention group, the AKI diagnosis and text module for AKI in the discharge letter of the index hospital stay were more frequently documented compared to those in the routine care group (40%/48% vs. 25%/34%, *p* = 0.034; *p* = 0.044, respectively, Figure 3). 

### 3.6. Independent Modifiers of Change in eGFR 

Continued intake of RAAS inhibitors (regression coefficient −7.42, *p* = 0.032) and presence of a cardiac device (regression coefficient −5.25, *p* = 0.037) were independently associated with a less pronounced decrease in eGFR from admission to the lowest value (Table 5). 

### 3.7. Other Patient Outcomes

**Figure 4:**
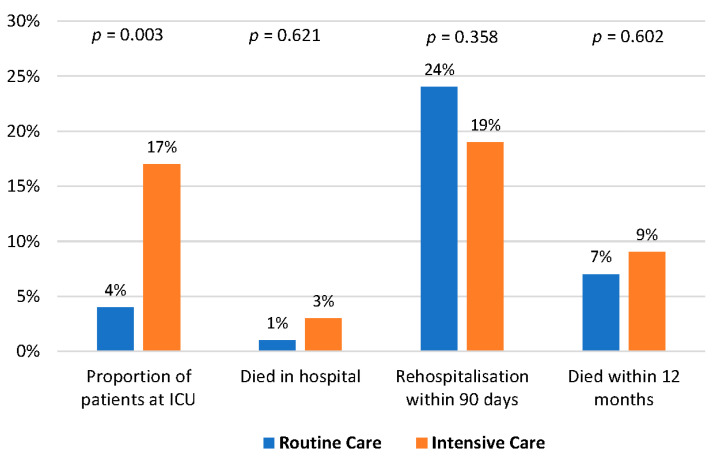
Patient-related outcome.

**Figure 5:**
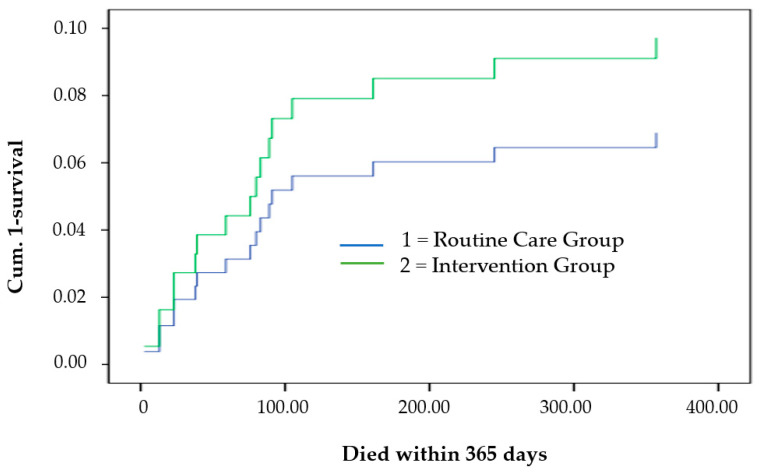
Survival analysis. COX’S proportional hazards regression model for 12-month mortality adjusting for acute coronary syndrome, atrial fibrillation, cardiac devices, and the use of ACE inhibitors or AT-1 blockers.

The rehospitalization rate within 90 days was 21.5% (24% in the routine care group vs. 19% in the intervention group), Figure 4. Sixteen patients died within 12 months, seven in the routine care group and nine in the intervention group. The proportion of patients developing MACE or requiring chronic renal replacement therapy within 90 days did not differ between both study groups (Figure 5).

### 3.8. Subgroup Analyses 

Excluding patients with hospital-acquired AKI, change of eGFR from hospital admission to three months after AKI did not differ between patients in the routine care group 9.0 (−3.6–18.5) mL/min/1.73 m^2^ versus patients in the intensive care group 7.0 (−4.7–22.2) mL/min/1.73 m^2^, *p* = 0.886. Also, three months after discharge, eGFR was similar in patients with community-acquired AKI in the routine care group 52.9 (33.5–66.8) mL/min/1.73 m^2^ compared to those in the intensive care group 50.0 (41.0–77.0) mL/min/1.73 m^2^), *p* = 0.647.

Also, posthoc analyses of other patient subgroups (patients with diabetes, patients aged > 70 years, female patients, and those with a cardiac device or using an ACE inhibitor/ AT-1 blocker) revealed no significant intervention effect on the primary study endpoint, Table 6 (all *p* > 0.05).

## 4. Discussion 

This study randomized two hundred cardiac patients to the intervention or routine care groups. The intervention consisted of an AKI electronic alert system combined with a care bundle, including education of physicians according to the KDIGO recommendations [10], medication intervention, and information about AKI to the patient, the attending physician, and the primary care physician. The primary study endpoint, loss of eGFR from admission to three months after AKI, did not differ between the study groups. Also, secondary endpoints, including loss of eGFR in hospital, proportions of patients with AKI-related complications, and magnitude of kidney recovery until three months after AKI, were comparable between the study groups. Proportions of patients receiving heart and kidney function-related interventions were similar in both groups. In the intervention group, the general physician was more frequently provided with more comprehensive information regarding AKI in the discharge letter. Finally, continued intake of RAAS inhibitors and the presence of a cardiac device were independently associated with a less pronounced decrease in eGFR from admission to the lowest value. 

Recent studies of AKI electronic alert and clinical decision support systems demonstrated variable results, which likely result from differences in study design, patient population, local context, and implementation strategies. Non-randomized studies evaluating AKI eAlerts enrolled heterogenous hospitalized patients, frequently used pre- and post-design, and reported a reduction of higher AKI stages, requirement of renal replacement therapy, length of stay, and in-hospital mortality [12,13,14]. Randomized controlled trials also included heterogeneous patient populations of hospitalized patients from all wards; however, they did not demonstrate patient benefit regarding mortality, renal replacement therapy requirement, or renal function recovery (Appendix A, Table A1). Most RCTs reported improvement in process-related parameters, including discontinuation of nephrotoxic medications, involvement of a nephrologist, and documentation in patient medical records [15]. 

Patients admitted to a cardiology ward may be different from patients admitted to other departments regarding etiology, timing, actionability, and recovery of AKI. Cardio-renal syndrome appears to be the major cause of AKI in cardiac patients, potentially requiring specific work-up and treatment [16,17]. None of the recent studies evaluated the impact of AKI electronic alert systems exclusively in a cardiac patient population. Therefore, a study investigating the efficacy of an AKI electronic alert system in patients exclusively admitted for cardiac diseases was needed. 

In this study, we observed a protective effect of RAAS inhibitors on the kidney. This is in line with experimental and clinical studies showing, in most cases, that RAAS inhibitors reduce proteinuria renal fibrosis, slow the decline of renal function, and protect against cardiovascular events. However, there are also data from an observational cohort study proposing that discontinuation of RAAS inhibitors in patients with advanced CKD may increase eGFR or slow its decline [18]. Findings of a recent multicenter randomized study assigning 411 patients with advanced CKD to discontinuation or continuation of RAAS inhibitors found that discontinuation was not associated with a significant between-group difference in the long-term rate of a decrease in the eGFR [19]. Also, a recent meta-analysis found that the continuation of RAAS inhibitors may benefit patients with CKD [20]. Overall, data on the use of RAAS inhibitors and the course of renal function suggest that renal function is protected, at least in the non-acute state. Also, we found an inverse association between the use of cardiac devices and eGFR decline. Cardiac implantable electronic devices may preserve central venous circulation and improve left ventricular function [21].

The present study reported the first-time impact of an AKI electronic alert system and care bundle on cardiac patients. Patient-related endpoints were not different from RCTs in general hospital patient populations. 

There may be several explanations why our intervention failed to demonstrate a patient benefit. In the present study, most patients developed AKI stage 1 or presented with community-acquired AKI with already recovering renal function during the days after admission, even without further intervention. Both factors may have diminished the chance of an intervention effect. Also, medication interventions used to treat AKI or cardio-renal syndrome on a cardiology ward were similar in both study groups except for the more frequent stop of antihypertensive medication in the intervention group, however, being a rare event with 6% of cases. Intake of RAAS inhibitors—being kidney protective also in this study—was continued as is current practice in many cardiology departments and may have reduced the intervention gradient between the study groups. 

In addition, patient characteristics slightly differed between study groups, with more patients presenting with acute coronary syndrome and fewer patients developing infections in the intervention group, potentially diminishing the intervention gradient. 

This study aimed to reduce selection bias using time-period clustered randomization and focused on a typical patient cohort with cardiorenal syndrome, which is widely spread among hospitalized patients. Patient follow-up was extended to 3 months for kidney function as recommended by KDIGO [10]. However, the study care bundle appeared to be not fully applied in the intervention group, including restriction of nephrology consultation to patients with severe AKI. Early nephrology consultation of patients with AKI may lead to better patient outcomes, as previously shown in a retrospective study by Meier et al. [22]. The lack of stratified patient randomization and monitoring of adherence regarding measures of the care bundle limited this study. Although we left the treating physicians unaware of whether the patient developed AKI during an intervention or control period, we acknowledge the potential carryover effect in treatment from the intervention to the control period as a study limitation.

The inclusion of patients with all stages of AKI inherently resulted in a greater proportion of patients with mild AKI versus those with severe AKI. Therefore, this study cannot exclude the effects of the study intervention in patients with severe AKI. Finally, we cannot exclude carryover effects from the intervention to the routine care group with measures of the study care bundle also applied in the control group. 

In sum, this study informs the cardiologist about the effects of an AKI electronic alert system in a typical cardiac patient cohort, separated for community and hospital-acquired AKI. 

Subsequent studies are preferred to be multicentric and may focus on patients with more severe AKI or routinely include nephrology consultation.

## 5. Conclusions

In this RCT, electronic alerts and a care bundle for AKI improved the process- but not patient-related endpoints in cardiac patients.

## Figures and Tables

**Table 1:** Patient baseline characteristics.

Variable	Routine Care Group (*n* = 100)	Intervention Group (*n* = 100)	*p*-Value
Demographics			
Age, years.	80 (70–84)	78 (66–84)	0.283
Female, *n*	44/100 (44%)	48/100 (48%)	0.670
BMI, kg/m^2^	27.5 (24.2–32.0)	27.6 (24.2–32.8)	0.621
Smoker, *n*	5/100 (5%)	13/100 (13%)	0.048
AKI grade			
1	89/100 (89%)	90/100 (90%)	0.844
2	9/100 (9%)	9/100 (9%)	
3	2/100 (2%)	1/100 (1%)	
AKI definition			
Hospital-acquired AKI			0.734
50% increase	26/100 (26%)	29/100 (29%)	
26.4 micromole/L increase	20/100 (20%)	16/100 (16%)	
Community-acquired AKI	54/100 (54%)	55/100 (55%)	
Type of admission			
Elective, *n*	55/99 (55%)	40/100 (40%)	0.028
Ambulance, *n*	27/99 (27%)	32/100 (32%)	0.465
Emergency Room, *n*	17/99 (17%)	28/100 (28%)	0.068
Level of care, grade >2, *n*	33/100 (33%)	33/100 (33%)	>0.99
Admission diagnosis			
TAVI, *n*	35/100 (35%)	25/100 (25%)	0.123
Acute coronary syndrome, *n*	15/100 (15%)	29/100 (29%)	0.017
Acute decompensated heart failure, *n*	16/100 (16%)	9/100 (9%)	0.135
Atrial fibrillation, *n*	9/100 (9%)	7/100 (7%)	0.602
Dyspnea, *n*	4/100 (4%)	8/100 (8%)	0.234
Lead explantation, *n*	5/100 (5%)	5/100 (5%)	>0.99
MitraClip, *n*	4/100 (4%)	2/100 (2%)	0.407
CRT-D implantation, *n*	2/100 (2%)	3/100 (3%)	0.651
Other, *n*	10/100 (10%)	12/100 (12%)	0.651
Comorbidities			
Type 2 diabetes (insulin), *n*	19/100 (19%)	18/100 (18%)	>0.99
Type 2 diabetes (oral medication), *n*	22/100 (22%)	28/100 (28%)	0.414
Arterial hypertension, *n*	76/100 (76%)	84/100 (84%)	0.216
Chronic kidney disease *, *n*	67/95 (70.5%)	65/97 (67.0%)	0.599
Hyperlipoproteinemia, *n*	29/100 (29%)	38/100 (38%)	0.231
Congestive heart disease, *n*	72/100 (72%)	75/100 (75%)	0.749
NYHA III, *n*	48/100 (48%)	35/100 (35%)	0.173
NYHA IV, *n*	5/100 (5%)	9/100 (9%)	0.154
LVEF, %	45.0 (30.3–55.0)	47.5 (31.5–60.0)	0.466
Peripheral vascular disease, *n*	8/100 (8%)	7/100 (7%)	>0.99
Atrial fibrillation, *n*	63/100 (63%)	45/100 (45%)	0.011
Pulmonary hypertension, *n*	8/100 (8%)	7/100 (7%)	>0.99
Chronic obstructive pulmonary disease, *n*	12/100 (12%)	18/100 (18%)	0.322
Asthma, *n*	4/100 (4%)	3/100 (3%)	>0.99
Previous myocardial infarction, *n*	14/100 (14%)	25/100 (25%)	0.050
NSTEMI, *n*	8/100 (8%)	15/100 (15%)	
STEMI, *n*	6/100 (6%)	10/100 (10%)	
Cardiac device, *n*	34/100 (34%)	32/100 (32%)	0.881
Coronary artery bypass graft, *n*	11/100 (11%)	13/100 (13%)	0.828
Stroke, *n*	13/100 (13%)	6/100 (6%)	0.091
TIA, *n*	1/100 (1%)	0/100 (0%)	>0.99
Depression, *n*	5/100 (5%)	4/100 (4%)	>0.99
Mental illness, *n*	3/100 (3%)	1/100 (1%)	0.621
Dementia, *n*	4/100 (4%)	2/100 (2%)	0.683
Current oncological disease, *n*	1/100 (1%)	5/100 (5%)	0.241
Thyroid disease, *n*	20/100 (20%)	13/100 (13%)	0.550
Arthrosis, *n*	12/100 (12%)	10/100 (10%)	0.651
Inflammatory joint disorders, *n*	0/100 (0%)	2/100 (2%)	0.497
Rheumatologic disease, *n*	5/100 (5%)	4/100 (4%)	>0.99
Osteoporosis, *n*	4/100 (4%)	5/100 (5%)	>0.99
Liver disease, *n*	5/100 (5%)	7/100 (7%)	0.767

BMI, body mass index; LVEF, left ventricular ejection fraction; STEMI, ST-segment elevation myocardial infarction; NSTEMI, non-ST-segment elevation myocardial infarction; TAVI, transcatheter aortic valve implantation; TIA, transient ischemic attack, * includes CKD stages 3 to 5.

**Table 2:** Medication during hospital stay.

Variable	Routine Care Group (*n* = 100)	Intervention Group (*n* = 100)	*p*-Value
Infection with antibiotics	30/100 (30%)	26/100 (26%)	0.529
Sepsis /septic shock	2/100 (2%)	0/100 (0%)	0.497
Antibiotics (including peri-interventional antibiotics), *n*	72/100 (72%)	45/100 (45%)	<0.001
Cephalosporin	47/100 (47%)	28/100 (28%)	0.006
Penicillin	15/100 (15%)	14/100 (14%)	>0.99
Tazobactam	7/100 (7%)	5/100 (5%)	0.552
Vancomycin	1/100 (1%)	2/100 (2%)	>0.99
Contrast media, *n*	87/100 (87%)	87/100 (87%)	>0.99
NSAID, *n*	9/100 (9%)	11/100 (11%)	0.620
Loop diuretics, *n*	87/100 (87%)	72/100 (72%)	0.009
Betablocker, *n*	83/100 (83%)	81/100 (81%)	0.713
ACE/AT-1 Blocker, *n*	77/100 (77%)	76/100 (76%)	>0.99
Statins, *n*	75/100 (75%)	66/100 (66%)	0.163
Aldosterone–Antagonists, *n*	46/100 (46%)	36/100 (36%)	0.151
Calcium-channel-blocker, *n*	32/100 (32%)	23/100 (23%)	0.154
Thiazide, *n*	8/100 (8%)	7/100 (7%)	0.788
Neprilysin-Inhibitor, *n*	7/100 (7%)	11/100 (11%)	0.323
Blood products, *n*	12/100 (12%)	12/100 (12%)	>0.99
Ezetimibe, *n*	11/100 (11%)	7/100 (7%)	0.323
NOAC, *n*	50/100 (50%)	41/100 (41%)	0.201
Platelet inhibitors, *n*	48/100 (48%)	52/100 (52%)	0.572
Metformin, *n*	15/100 (15%)	15/100 (15%)	>0.99
SGLT2 inhibitors, *n*	10/100 (10%)	8/100 (8%)	0.621
Insulin, *n*	18/100 (18%)	17/100 (17%)	0.852

ACE, angiotensin-converting enzyme; AT-1, angiotensin-1; NOAC, novel oral anticoagulant; NSAID, nonsteroidal anti-inflammatory drug; SGLT2, sodium–glucose co-transporter 2.

**Table 3:** Patient-related interventions.

Variable	Routine Care Group (*n* = 100)	Intervention Group (*n* = 100)	*p*-Value
At least one patient-related intervention	28/100 (28%)	33/100 (33%)	0.443
Multiple patient-related interventions	5/100 (5%)	5/100 (5%)	>0.99
Interventions			
Fluid administration	20/100 (20%)	21/100 (21%)	0.861
Stop nephrotoxic medication	9/100 (9%)	6/100 (6%)	0.421
Stop antihypertensive medication during hypotensive period	0/100 (0%)	6/100 (6%)	0.029
Adjust diuretics	1/100 (1%)	5/100 (5%)	0.212
Nephrology consultation	1/100 (1%)	0/100 (0%)	>0.99
Initiation RRT	2/100 (2%)	0/100 (0%)	0.497

RRT, renal replacement therapy.

**Table 4:** Patient outcomes.

Variable	Routine Care Group (*n* = 100)	Intervention Group (*n* = 100)	*p*-Value
Renal Outcome			
Serum creatinine, µmol/L			
at admission	121.0 (93.0–160.0)	109.0 (86.0–142.5)	0.117
peak	157.0 (118.0–226.0)	142.0 (105.5–202.0)	0.206
at day	2 (0–8)	3 (0–6)	0.825
Delta admission—peak	33.0 (0.0–61.0)	27.0 (0.0–67.0)	0.943
at discharge	99.0 (71.8–128.3)	92.0 (72.5–131.5)	0.689
3 months after AKI	112.0 (84.0–140.0)	104.0 (82.0–148.0)	0.642
∆ admission—3 months after AKI	6.4 (−13.8–33.8)	3.5 (−27.4–25.3)	0.206
∆ discharge—3 months	−5.5 (−25.9–53.3)	−5.0 (−22.3–31.0)	0.858
eGFR, mL/min/1.73 m^2^			
at admission	44.0 (28.0–66.0)	51.0 (35.0–68.0)	0.190
lowest	33.0 (22.0–44.0)	36.5 (24.0–50.75)	0.197
∆ admission—lowest	−8.0 (−22.0–[−1.0])	−9.0 (−24.0–[−1.0])	0.586
at discharge	63.2 (44.1–84.6)	67.8 (41.7–83.1)	0.836
3 months after AKI	49.1 (33.0–64.3)	47.0 (35.0–73.5)	0.770
∆ admission—3 months after AKI	−0.5 (−7.6–10.8)	1.0 (−13.5–15.1)	0.527
∆ discharge—3 months	−12.7 (−27.2–[−2.0])	−14.6 (−26.1–[−1.8])	0.861
AKI-related complications *	6/100 (6%)	9/100 (9%)	0.421
Process-related endpoints			
AKI diagnosis in discharge letter, *n*	25/100 (25%)	40/100 (40%)	0.034
Text module for AKI in discharge letter, *n*	34/100 (34%)	48/100 (48%)	0.044
Cause of AKI in discharge letter, *n*	26/100 (26%)	29/100 (29%)	0.752
AKI cause, *n*			
Pre-renal	92 (92%)	83/100 (83%)	0.055
Intra-renal	7/100 (7%)	15/100 (15%)	
Post-renal	1/100 (1%)	2/100 (2%)	
Recommendation for outpatient follow-up, *n*	54/100 (54%)	61/100 (61%)	0.391
Outcome			
Proportion of patients at ICU, *n*	4/100 (4%)	17/100 (17%)	0.003
Length of stay in hospital, days	11.0 (7.0–18.0)	10.5 (6.0–15.8)	0.518
RRT during hospital stay, *n*	3/100 (3%)	1/100 (1%)	0.621
Died in hospital, *n*	1/100 (1%)	3/100 (3%)	0.621
Discharge			
home, *n*	85/100 (85%)	84/100 (84%)	>0.99
nursing home. *n*	0/100 (0%)	2/100 (2%)	0.497
rehab, *n*	15/100 (15%)	15/100 (15%)	>0.99
external hospital, *n*	11/100 (11%)	7/100 (7%)	0.459
Follow-up			
Rehospitalization within 90 days	24/100 (24%)	19/100 (19%)	0.358
MACE within 90 days	1/100 (1%)	1/100 (1%)	>0.99
Chronic RRT within 90 days	3/100 (3%)	4/100 (4%)	0.722
Died within 12 months	7/100 (7%)	10/100 (10%)	0.447

MACE, major adverse cardiac events; RRT, renal replacement therapy; * Hyperkalemia, pulmonary edema, and renal acidosis.

**Table 5:** Multivariable linear regression analysis for eGFR decrease from admission to lowest value.

Variable	Regression Coefficient	95% CI (Lower to Upper Limit)	*p*-Value
ACE inhibitor/AT-1 blocker	−7.69	−14.47 to −0.90	0.027
Cardiac device	−5.21	−10.14 to −0.28	0.039
IDDM	3.86	−2.31 to 10.0	0.218
Sacubitril/Valsartan	−5.78	−15.72 to 4.17	0.253
Intervention	2.15	−2.58 to 6.87	0.370
Age	−0.09	−0.34 to 0.16	0.488
LVEF (%)	−0.05	−0.22 to 0.12	0.591
Loop diuretics	−0.53	−7.10 to 6.04	0.873

IDDM, insulin-dependent diabetes; LVEF, left ventricular ejection fraction.

**Table 6:** Analyses of potential intervention effects of the primary study endpoint in subgroups of patients.

Subgroup	Routine Care Group	Intervention Group	*p*-Value
	∆ eGFR, ml/min/1.73 m^2^ (admission to 3 months after AKI)	∆ eGFR, ml/min/1.73 m² (admission to 3 months after AKI)	
Diabetes	−3.0 (−12.0–8.0)	4.5 (−8.3–21.0)	0.419
Age > 70 years	−1.5 (−7.9–8.0)	−5.5 (−14.8–7.0)	0.348
Cardiac device	9.5 (−3.8–14.0)	0.0 (−13.0–10.0)	0.067
Female	−2.6 (−11.5–8.6)	−5.5 (−15.8–6.0)	0.323
ACE inhibitor/AT-1 blocker	−1.0 (−7.6–10.0)	−2.2 (−15.7–16.0)	0.398

## Data Availability

The data that support the findings of this study are available upon request from the corresponding author (AHF).

## Appendix A

**Table A1:** Recent clinical studies regarding hospital AKI-e-alert.

Reference	Study Design	Patients (n =)	AKI Definition Used for e-Alert *	Inclusion Criteria	Main Result	Process Parameter Reported	PMID
Atia, J. et al. 2023 [6]	prospective clinical study (before and after design)	17,433	KDIGO	>18 years, inpatient diagnosed with AKI	After the introduction of the e-alert, progression to higher AKI stage, emergency readmission to hospital, and death during admission were significantly reduced.	More prescriptions were stopped for drugs that adversely affect renal function in AKI	36647011
Kotwal, S. et al. 2023 [8]	prospective clinical study	639	KDIGO	>18 years, inpatient diagnosed with AKI	AKI eAlert bundle reduced LOS in patients with AKI stage 1	documentation of AKI better in intervention group (94.8% vs. 83.4%; *p* = 0.001), with higher rates of nephrology consultation (25% vs. 19%; *p* = 0.04), cessation of nephrotoxins (25.3 vs. 18.8%; *p* = 0.045)	35438795
Wilson, F.P. et al. 2023 [15]	RCT	5060	KDIGO	>18 years, inpatient diagnosed with AKI and active order for one or more of the three medications of interest	a composite of progression of AKI, RRT, or death within 14 days—occurred in 585 (23.1%) of individuals in the alert group and 639 (25.3%) of patients in the usual care group (RR 0.92, 0.83–1.01, *p* = 0.09)	medication of interest was discontinued in 61.1% of the alert group vs. 55.9% of the usual care group (RR 1.08, 1.04–1.14, *p* = 0.0003)	37198160
Shi, Y. et al. 2022 [23]	secondary analysis of a multicenter RCT	6030	KDIGO	>18 years, inpatient diagnosed with AKI	Inconclusive results	/	36466503
Thanapongsatorn, P. et al. [24]	RCT	98	KDIGO	>18 years, who had survived from AKI 2–3, as defined by the KDIGO	eGFR at 12 months was comparable between the two groups (66.74 vs. 61.12 mL/min/1.73 m^2^, *p* = 0.54), urine albumin: creatinine ratio was lower in the comprehensive care group (36.83 vs. 177.70 mg/g, *p* = 0.036)	Compared to the standard care group, the comprehensive care group had better feasibility outcomes; 3 days dietary record, drug reconciliation, and drug alerts (p < 0.001).	34465357
Thomas, M.E. et al. 2021 [7]	Observational cohort study	1762	KDIGO	>18 years, with an alert (Stages 1–3) due to AKI detected from a serum creatinine	low rates of death within 30 days (11–15%) or requirement for RRT (0.4–3.7%) were seen.	A median of 3.0 non-medication recommendations and 0.5 medication-related recommendations per patient were made by the outreach team a median of 15.7 h after the AKI alert.	31860096
Haase-Fielitz, A. et al. 2020 [11]	RCT	52	KDIGO	Patients with AKI, age > 18	GFR went down, from hospital admission to discharge, by 3 mL/min/1.73 m^2^ (1st–3rd quartile: (6–20)) in the intervention group and by 13 mL/min/1.73 m^2^ in the control group (1st–3rd quartile: [0; −25]; *p* = 0.09). Complications of AKI were rarer in the intervention group (15% vs. 39%; *p* = 0.03).	in the intervention group, cause of AKI was identified more frequently (27% vs. 4%; *p* = 0.05); drugs with relevance to the kidney were discontinued more frequently (65% vs. 31%; *p* = 0.01); and AKI diagnosis was more frequently documented in the patient’s chart (58% vs. 37%; *p* = 0.03)	32530412
Selby, N. et al. 2019 [9]	Multicenter, stepped-wedge cluster RCT	24,059 AKI episodes	KDIGO	Patients with AKI, age > 18 years, who were hospitalized for at least one night during the study period	The intervention did not reduce 30-day mortality but did reduce hospital length of stay.	Improvement of quality of care	31058607
Wu, Y. et al. 2018 [25]	RCT	875	KDIGO	>18 years, no CKD or chronic RRT, kidney transplantation, amputation, or clinical evidence to support a diagnosis of AKI, baseline sCr < 353.6 μmol/L	The sensitivity, specificity, Youden Index, and accuracy of the AKI e-alert system were 99.8, 97.7, 97.5 and 98.1%, respectively	prevalence of nephrology consultation in the e-alert group was higher than that in the non-e-alert group (9.0 and 3.7%, *p* = 0.001)	29556903
Hodgson, L.E. et al. 2018 [26]	controlled before-and-after study	30,295	KDIGO	patients with AKI and stayed at least one night	incidence of HA-AKI reduced (odds ratio 0.990, 95% CI 0.981–1.000, *p* = 0.049)	process measures significantly improved at the intervention site.	30089118
Biswas, A. et al. 2018 [27]	Secondary analysis of a clinical trial	2278	KDIGO	Adults who developed at least stage 1 AKI as defined by KDIGO	effect of targeting alerts to patients with higher scores: in the high uplift group, alerting was associated with a reduction in change in creatinine of −5.3% (*p* = 0.03)	/	29599299
Wilson, F.P. et al. 2015 [28]	RCT	1201	KDIGO	Patients with AKI, age > 18	Composite relative maximum change in creatinine, RRT, and death at 7 days did not differ between the alert group and the usual care group (*p* = 0.88).	/	25726515

AKI, acute kidney injury; RRT, renal replacement therapy; LOS, length of stay in hospital. * All studies used a (modified) KDIGO-guideline-related AKI care bundle.

**Table A2:** Laboratory parameters.

Variable	Routine Care (*n* = 100)	Intervention Group (*n* = 100)	*p*-Value
Laboratory markers at admission			
Serum creatinine, µmol/L	121.0 (93.0–160.0)	109.0 (86.0–142.5)	0.117
eGFR, ml/min/1.73 m^2^	44.0 (28.0–66.0)	51.0 (35.0–68.0)	0.190
Urea, mmol/L	10.6 (7.3–15.1)	9.3 (6.9–14.0)	0.350
Glucose, mmol/L	7.2 (5.8–9.8)	7.7 (6.4–10.9)	0.123
Hemoglobin, mmol/L	7.8 (7.1–8.3)	7.9 (6.9–11.8)	0.813
Leukocytes, per nL	8.5 (6.6–10.3)	8.8 (7.0–11.8)	0.144
Platelets, per nL	218 (158–254)	194 (151–271)	0.583
NT-proBNP, pg/mL	3208 (1321–11.128)	2942 (645–6616)	0.057
CRP, mg/L	8.9 (3.4–34.0)	6.2 (2.5–16.6)	0.149
Potassium, mmol/L	4.6 (4.2–5.0)	4.4 (4.1–4.8)	0.156
pH	7.40 (7.35–7.43)	7.40 (7.32–7.45)	0.971
Bicarbonate, mmol/L	24.3 (20.5–26.4)	23.1 (19.9–27.1)	0.579
BE, mmol/L	−0.05 (−4.3–1.8)	−0.8 (−4.9–3.4)	0.937
Laboratory markers at AKI Alarm Day			
Serum creatinine, µmol/L	116.5 (78.5–157.0)	109.5 (70.0–167.5)	0.514
Urea, mmol/L	10.6 (6.9–16.8)	8.4 (6.1–17.0)	0.325
Glucose, mmol/L	6.8 (5.6–8.9)	6.8 (6.1–8.5)	0.768
Hemoglobin, mmol/L	6.9 (6.0–7.9)	7.2 (6.4–8.2)	0.158
Leukocytes, per nL	9.3 (7.3–10.9)	8.6 (6.9–10.5)	0.248
Platelets, per nL	177 (134–247)	177 (138–244)	0.897
NT-proBNP, pg/mL	9150 (1724–33292)	2530.5 (1191.7–8897.5)	0.272
CRP, mg/L	35.0 (12.8–85.8)	23.9 (9.8–57.2)	0.215
Potassium, mmol/L	4.2 (3.9–4.6)	4.1 (3.8–4.5)	0.758
pH	7.43 (7.39–7.46)	7.44 (7.40–7.46)	0.637
Bicarbonate, mmol/L	25.6 (23.5–28.6)	26.3 (24.1–29.1)	0.578
BE, mmol/L	1.5 (−0.7–3.9)	1.85 (−0.5–4.0)	0.520

## References

[1] Hoste E.A.J., Kellum J.A., Selby N.M., Zarbock A., Palevsky P.M., Bagshaw S.M., Goldstein S.L., Cerdá J., Chawla L.S. (2018). Global epidemiology and outcomes of acute kidney injury. Nat. Rev. Nephrol..

[2] Sawhney S., Fluck N., Fraser S.D., Marks A., Prescott G.J., Roderick P.J., Black C. (2016). KDIGO-based acute kidney injury criteria operate differently in hospitals and the community-findings from a large population cohort. Nephrol. Dial. Transplant..

[3] Prescott A.M., Lewington A., O’Donoghue D. (2012). Acute kidney injury: Top ten tips. Clin. Med..

[4] Siew E.D., Parr S.K., Wild M.G., Levea S.L., Mehta K.G., Umeukeje E.M., Silver S.A., Ikizler T.A., Cavanaugh K.L. (2019). Kidney Disease Awareness and Knowledge among Survivors ofAcute Kidney Injury. Am. J. Nephrol..

[5] https://www.england.nhs.uk/2014/06/psa-aki/.

[6] Atia J., Evison F., Gallier S., Hewins P., Ball S., Gavin J., Coleman J., Garrick M., Pankhurst T. (2023). Does acute kidney injury alerting improve patient outcomes?. BMC Nephrol..

[7] Thomas M.E., Abdelaziz T.S., Perkins G.D., Sitch A.J., Baharani J., Temple R.M. (2021). The Acute Kidney Outreach to Prevent Deterioration and Death trial: A large pilot study for a cluster-randomized trial. Nephrol. Dial. Transplant..

[8] Kotwal S., Herath S., Erlich J., Boardman S., Qian J., Lawton P., Campbell C., Whatnall A., Teo S., Horvath A.R. (2023). Electronic alerts and a care bundle for acute kidney injury-an Australian cohort study. Nephrol. Dial. Transplant..

[9] Selby N.M., Casula A., Lamming L., Stoves J., Samarasinghe Y., Lewington A.J., Roberts R., Shah N., Johnson M., Jackson N. (2019). An Organizational-Level Program of Intervention for AKI: A Pragmatic Stepped Wedge Cluster Randomized Trial. J. Am. Soc. Nephrol..

[10] Kellum J.A., Lameire N., Aspelin P., Barsoum R.S., Burdmann E.A., Goldstein S.L., Herzog C.A., Joannidis M., Kribben A., Levey A.S. (2012). Kidney disease: Improving global outcomes (KDIGO) acute kidney injury work group. KDIGO clinical practice guideline for acute kidney injury. Kidney Int. Suppl..

[11] Haase-Fielitz A., Elitok S., Schostak M., Ernst M., Isermann B., Albert C., Robra B.P., Kribben A., Haase M. (2020). The Effects of Intensive Versus Routine Treatment in Patients with Acute Kidney Injury. Dtsch. Arztebl. Int..

[12] Tome A.C.N., Ramalho R.J., Dos Santos K.F., Ponte B., Agostinho H., Machado M.N., Lopes M.B., Abbud-Filho M., de Lima E.Q. (2022). Impact of an Electronic Alert in Combination with a Care Bundle on the Outcomes of Acute Kidney Injury. Diagnostics.

[13] Ebah L., Hanumapura P., Waring D., Challiner R., Hayden K., Alexander J., Henney R., Royston R., Butterworth C., Vincent M. (2017). A Multifaceted Quality Improvement Programme to Improve Acute Kidney Injury Care and Outcomes in a Large Teaching Hospital. BMJ Qual. Improv. Rep..

[14] Kolhe N.V., Staples D., Reilly T., Merrison D., Mcintyre C.W., Fluck R.J., Selby N.M., Taal M.W. (2015). Impact of Compliance with a Care Bundle on Acute Kidney Injury Outcomes: A Prospective Observational Study. PLoS ONE.

[15] Wilson F.P., Yamamoto Y., Martin M., Coronel-Moreno C., Li F., Cheng C., Aklilu A., Ghazi L., Greenberg J.H., Latham S. (2023). A randomized clinical trial assessing the effect of automated medication-targeted alerts on acute kidney injury outcomes. Nat. Commun..

[16] Jentzer J.C., Bihorac A., Brusca S.B., Del Rio-Pertuz G., Kashani K., Kazory A., Kellum J.A., Mao M., Moriyama B., Morrow D.A. (2020). Contemporary Management of Severe Acute Kidney Injury and Refractory Cardiorenal Syndrome: JACC Council Perspectives. J. Am. Coll. Cardiol..

[17] Di Lullo L., Bellasi A., Russo D., Cozzolino M., Ronco C. (2017). Cardiorenal acute kidney injury: Epidemiology, presentation, causes, pathophysiology and treatment. Int. J. Cardiol..

[18] Ahmed A.K., Kamath N.S., El Kossi M., El Nahas A.M. (2010). The impact of stopping inhibitors of the renin-angiotensin system in patients with advanced chronic kidney disease. Nephrol. Dial. Transplant..

[19] Bhandari S., Mehta S., Khwaja A., Cleland J.G.F., Ives N., Brettell E., Chadburn M., Cockwell P., STOP ACEi Trial Investigators (2022). Renin-Angiotensin System Inhibition in Advanced Chronic Kidney Disease. N. Engl. J. Med..

[20] Naveed H., Tirumandyam G., Krishna Mohan G.V., Gul S., Ali S., Siddiqui A., Suarez Z.K., Khan A. (2023). Effect of Discontinuation of Renin Angiotensin-System Inhibitors in Patients with Advanced Chronic Kidney Disease: A Meta-Analysis. Cureus.

[21] Adelstein E.C., Shalaby A., Saba S. (2010). Response to cardiac resynchronization therapy in patients with heart failure and renal insufficiency. Pacing Clin. Electrophysiol..

[22] Meier P., Bonfils R.M., Vogt B., Burnand B., Burnier M. (2011). Referral patterns and outcomes in noncritically ill patients with hospital-acquired acute kidney injury. Clin. J. Am. Soc. Nephrol..

[23] Shi Y., Wang H., Bai L., Wu Y., Zhang L., Zheng X., Lv J.H., Pei H.H., Bai Z.H. (2022). The rate of acute kidney injury (AKI) alert detection by the attending physicians was associated with the prognosis of patients with AKI. Front. Public Health.

[24] Thanapongsatorn P., Chaikomon K., Lumlertgul N., Yimsangyad K., Leewongworasingh A., Kulvichit W., Sirivongrangson P., Peerapornratana S., Chaijamorn W., Avihingsanon Y. (2021). Comprehensive versus standard care in post-severe acute kidney injury survivors, a randomized controlled trial. Crit. Care.

[25] Wu Y., Chen Y., Li S., Dong W., Liang H., Deng M., Chen Y., Chen S., Liang X. (2018). Value of electronic alerts for acute kidney injury in high-risk wards: A pilot randomized controlled trial. Int. Urol. Nephrol..

[26] Hodgson L.E., Roderick P.J., Venn R.M., Yao G.L., Dimitrov B.D., Forni L.G. (2018). The ICE-AKI study: Impact analysis of a Clinical prediction rule and Electronic AKI alert in general medical patients. PLoS ONE.

[27] Biswas A., Parikh C.R., Feldman H.I., Garg A.X., Latham S., Lin H., Palevsky P.M., Ugwuowo U., Wilson F.P. (2018). Identification of Patients Expected to Benefit from Electronic Alerts for Acute Kidney Injury. Clin. J. Am. Soc. Nephrol..

[28] Wilson F.P., Shashaty M., Testani J., Aqeel I., Borovskiy Y., Ellenberg S.S., Feldman H.I., Fernandez H., Gitelman Y., Lin J. (2015). Automated, electronic alerts for acute kidney injury: A single-blind, parallel-group, randomised controlled trial. Lancet.

